# Malnutrition-inflammation is a risk factor for cerebral small vessel diseases and cognitive decline in peritoneal dialysis patients: a cross-sectional observational study

**DOI:** 10.1186/s12882-017-0777-1

**Published:** 2017-12-20

**Authors:** Ke Zheng, Haiyun Wang, Bo Hou, Hui You, Jing Yuan, Kai Luo, Limeng Chen, Mingxi Li, Qun Xu, Yicheng Zhu, Liying Cui, Sagar Uday Nigwekar, Feng Feng, Xuemei Li

**Affiliations:** 1 Department of Nephrology, Peking Union Medical College Hospital, Chinese Academy of Medical Sciences, No.1 Shuaifuyuan, Dongcheng District, Beijing, 100730 People’s Republic of China; 2 Department of Radiology, Peking Union Medical College Hospital, Chinese Academy of Medical Sciences, Beijing, People’s Republic of China; 3 Department of Neurology, Peking Union Medical College Hospital, Chinese Academy of Medical Sciences, Beijing, People’s Republic of China; 4 Department of Epidemiology and biostatistics, Institute of basic medical sciences, Chinese Academy of Medical Sciences, Beijing, People’s Republic of China; 5 Division of Nephrology, Department of Medicine, Massachusetts General Hospital, Boston, USA

**Keywords:** Peritoneal dialysis, Cerebral small vessel disease, Cognitive function, Malnutrition, Inflammation

## Abstract

**Background:**

Chronic kidney disease patients have an increased prevalence of subclinical cerebrovascular diseases. Dialysis patients have severe vascular diseases burden. The cerebral small vessel diseases (CSVD) are difficult to find by clinical assessment. The evaluation of CVSD needs MRI. Cognitive impairment is a consequence of CVSD which is diagnosed by cognitive testing. These limited the study of CVSD and cognitive function in dialysis patients. Peritoneal dialysis (PD) patients are minority of dialysis population. We know even fewer about the CVSD in this special population.

**Methods:**

In this cross-sectional study, we enrolled 72 PD patients who received care at the Peking Union Medical College hospital peritoneal dialysis center. CSVD were assessed by brain MR images. Cognitive function was evaluated with the Chinese version of the MMSE and MoCA.

**Results:**

In our PD patients, the brain MRI showed the prevalence different signs of CSVD were: lacunar infarcts 38.9%, microbleeds 36.1%, abnormal brain white matter hyperintensities (WMHs) 48.6%, and intracerebral hemorrhage 4.2%. 25% and 86.8%of our patients could be diagnosed as cognitive impairment, according to the MMSE and MoCA test, respectively. nPCR was lower in patients with a lacunar infarct or intracerebral hemorrhage, and relative to the MMSA/MoCA score; hsCRP was higher in patients with lacunar infarct or abnormal WMHs and negative relative to the MMSA/MoCA score. In logistic regression analyses, nPCR was an independent risk factor for lacunar infarcts and impaired cognitive function. The presence of lacunar infarct was an independent risk factor for cognitive function decline.

**Conclusion:**

We demonstrated a high prevalence of CSVD and cognitive impairment in our PD patients. Lacunar infarct was the main kind of CVSD responsible for PD patients cognitive function decline. Our novel observation also revealed an association between malnutrition-inflammation and CSVD.

**Electronic supplementary material:**

The online version of this article (10.1186/s12882-017-0777-1) contains supplementary material, which is available to authorized users.

## Background

CKD patients have an increased prevalence of subclinical cerebrovascular diseases, including silent brain infarcts (SBIs), white matter magnetic resonance hyperintensities (WMHs), and cerebral microbleeds [1].

Most SBIs are lacunar infarcts [2]. Lacunar infarcts, WMHs and microbleeds are signs of cerebral small vessel disease (CSVD). Dialysis patients have more severe vascular disease burden than non-dialysis CKD patients. Therefore, it is reasonable to deduce that there would be a high prevalence of CSVD in dialysis patients. However, CSVDs are difficult to detect with clinical evaluation in our daily practice. For such subclinical lesions, we need brain magnetic resonance (MR) images to identify them. This limits the study of CSVD in dialysis population. A few small sample studies evaluated hemodialysis patients’ brain lesions by MR images [3, 4]. Peritoneal dialysis (PD) is a different renal replacement modality comparing to hemodialysis, which induces less hemodynamic changes, but raises more metabolic disorders. PD patients are minority of dialysis population in most of western countries. For this group of patients, there are very limited data about their CSVD.

Cognitive impairment (CI) is clinical sequence of CSVD. CI raises patient safety concerns and limits their ability to understand medical advice and their decision-making ability. SBIs and WMHs are predictive of cognitive decline and dementia in CKD patients [5]. In hemodialysis patients, the reported ratio of CI was 30–60% [3, 6], which was 2-fold higher than that of the age-matched healthy subjects. In PD patients, like their CSVD, we know few about their cognitive function changes.

Dialysis patients have more complex comorbidity related to vascular disease than non-CKD and CKD non-dialysis patients. The pathogenic mechanism of CSVD and CI in dialysis patients is still not well understood.

In this cross-sectional study, we investigated our PD patients’ CSVD by brain MR images and their cognitive status by MMSE and MoCA questionnaires. We also tried to determine the possible pathogenic mechanism of CSVD and brain functional changes in this study.

## Methods

### Study design

This was a cross-sectional study. We recruited PD patients from the Peking Union Medical College Hospital dialysis center. The study protocol was approved by the institutional review board of Peking Union Medical College Hospital. All subjects gave written informed consent to participate in the study prior to the initiation of the study.

### Subjects

From Jul 2013 to Jul 2014, 196 convenient subjects who were on peritoneal dialysis in the Peking Union Medical College Hospital dialysis center were screened. The inclusion criteria were age of 17–80 yrs. and chronic peritoneal dialysis dependence for at least 1 month. Subjects who met the following criteria were excluded from the study: 1). Conditions that interfered with finishing the interviews and examinations, including blindness, deafness, difficulty in upper limb movements, poor general status, etc.; 2). Metabolic encephalopathy, such as hepatic encephalopathy, uremic encephalopathy, and hypoxic encephalopathy; 3). History of mental disorder, emotional disorder, and epilepsy on long-term treatment; 4). Diagnosis of dementia; 5). Patients with a history of non-atherosclerosis arterial disease (e.g., Takayasu arteritis); 6). Patients with addiction to drugs or alcohol; and 7). Refusal to participate in the study.

### Clinical data collection

We collected information about patients’ medical history (including history of hypertension, diabetes mellitus, atherosclerosis and stroke), demographic data, body mass index (BMI), blood pressure, weekly Kt/V and normalized protein catabolic rate (nPCR). We also collected patients’ blood examination results from our clinical practice, which were near to the date of cognitive and MR evaluation, including hemoglobin, serum creatinine (SCr), blood urea nitrogen (BUN), serum albumin, lipid profiles, high sensitivity C reactive protein (hs-CRP), serum calcium and phosphorus ferritin, transferrin saturation (TSAT), β2-MG, and intact PTH (iPTH). The patients’ disease history and medication were evaluated at the same time.

### Brain MR imaging

Patients underwent brain MR examination on a 3.0 T MR scanner (DISCOVERY MR750; General Electric, Milwaukee, WI), which underwent daily quality assurance monitoring with a head 8-channel phased array coil operated by research-dedicated technical staff. Imaging sequences included T1-weighted and T2-weighted sequences, fluid-attenuated inversion recovery (FLAIR) and susceptibility weighted imaging (SWI). The imaging plane of all sequences was axial images parallel to the brain bicommissural line.

### MRI analysis

All MRIs were independently assessed by two experienced neuroradiologists who were blinded to the clinical information. MR images were assessed for the presence and location of different signs of CSVD, including recent or chronic lacunar infarcts, microbleeds, WMHs and intracerebral hemorrhage. If there was a discrepancy, consensus was reached after discussion.

The definition of recent lacunar infarct was a lesion that should be less than 20 mm in its maximum diameter in the axial plane with restricted diffusion. Chronic lacunar infarcts were defined as deep gray matter lesions or lesions in the white matter with cavitation (signal similar to cerebral spinal fluid) of between 3 mm and 15 mm in diameter, which is similar to the definition of lacunae of presumed vascular origin [7].

Microbleeds were defined as small (2–10 mm), homogeneous, round foci of low signal intensity on gradient echo images in the cerebellum, brainstem, basal ganglia, thalami, deep white matter, or cortico-subcortical junction. Lesion larger than 10 mm were defined as chronic intracerebral hemorrhage.

Deep and periventricular WMHs were graded according to the Fazekas scale from 0 to 3; the total Fazekas score was the sum of these two parts (0 to 6) [8]. “Abnormal WMHs” was defined as a total Fazekas score > 2.

### Cognitive evaluation

Eight interviewers consisting of dialysis staff and nurses in our dialysis centers were trained by neurologist to complete the cognitive tests. At the completion of training, the inter-rater reliability of the cognitive tests was assessed by rating a videotaped model interview.

The Chinese version of the Mini Mental State Examination (MMSE) [9, 10] and the Chinese version of Montreal Cognitive Assessment (MoCA) [11, 12] were administered for the cognitive function evaluation.

The cut-off points of the MMSE score was ≤19 for illiterate subjects, ≤22 for subjects with education less than 7 years (primary school) and ≤26 for subjects with more than 7 years of education (middle school and above). The cut-off point for the MoCA score was ≤26. For subjects with less than 12 years of education, one more score was added to the crude MoCA score for educational adjustment.

### Data collection and statistical analysis

Study data were collected and managed using REDCap electronic data capture tools hosted at Peking Union Medical College Hospital.

Continuous data were presented as the mean ± standard deviation, except for the variables that followed the log-normal distribution, such as hs-CRP, nPCR, TSAT, MMSE and MoCA, which were presented as the log-transform mean ± standard deviation. For those with high skew, the median and interquartile range were used as basic description metrics. Categorical data were presented as proportions. One-way analysis of variance, Kruskal-Wallis, Chi-square test and fisher exact test were used to compare the differences between different CSVD sign groups. Pearson correlation analyses were performed to explore the relationships between continuous variables and cognition scores, while analysis of variance (ANOVA) was used for association between category variables and the recognition score in the preliminary analysis.

Univariate and multivariable logistic regression models were built to examine the association between demographics and clinical indicators as well as the CSVD signs with the CSVD signs as the dependent variables. Those with statistically significant coefficients in the univariate models were selected as the candidate variables for the multivariable analyses. Specifically, only the interest indicators were included in the basic models. Then, we built a series of multivariate models with the inclusion of the interest variables and candidate variables after adjusting for basic information, such as the age, gender, drinking and smoking status. Additionally, we used the general linear model to explore the relationships between basic information (age, gender, smoking, etc.), CSVD signs and cognition scores (MMSE and MoCA) with the log transform of MMSE and MoCA as the dependent variables. For this part, univariate models were first built to select the candidate variables; then, we performed the multivariate analyses with the selected and interest variables, such as the CSVD signs, after adjusting for the age, gender, smoking status, etc.

All analyses were performed with the SAS 9.4 (SAS Institute’s Inc., Cary. NC. USA.) and statistical significance was defined as a *p* value less than 0.05 (two tails).

## Results

**Fig. 1:**
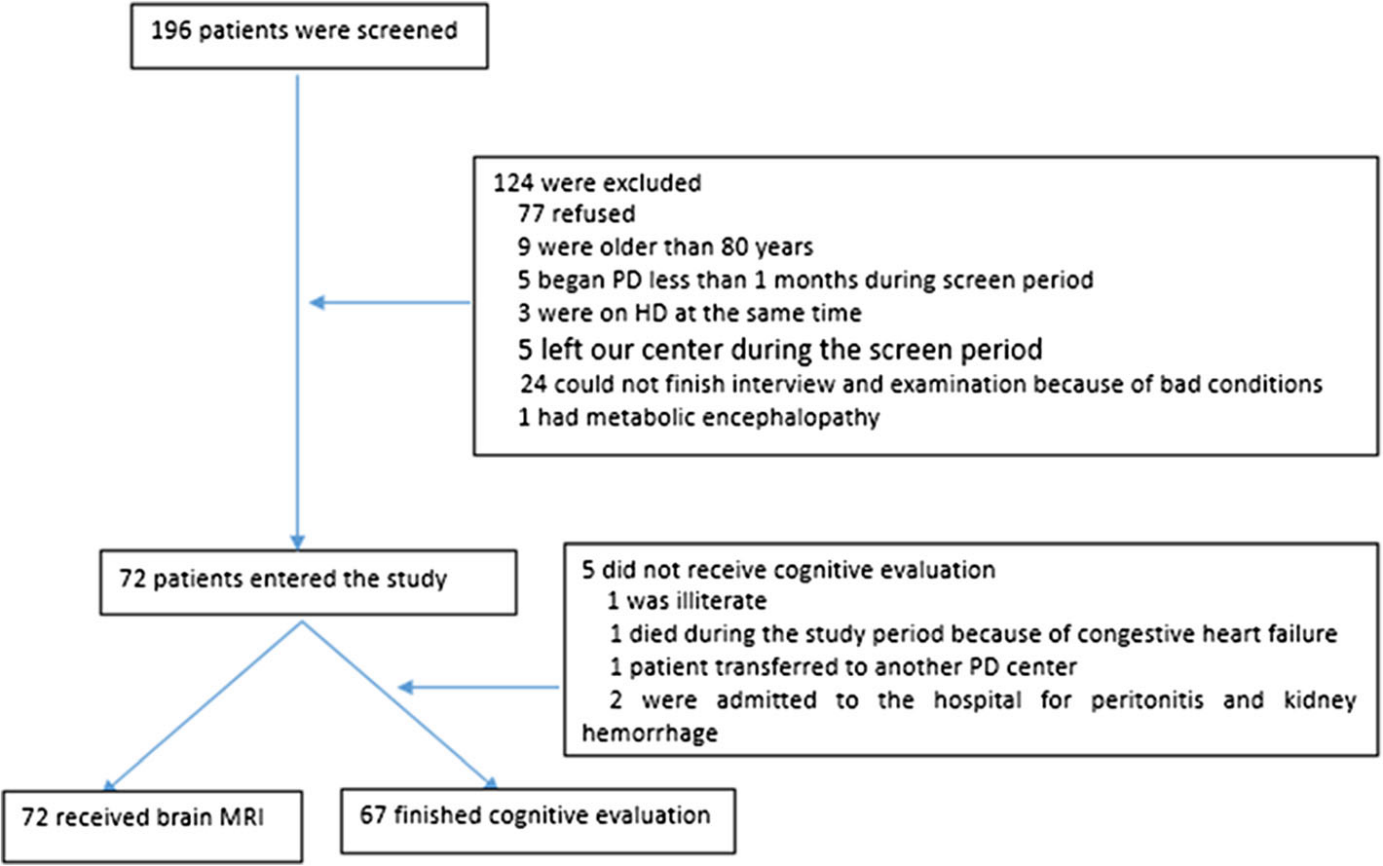
Flow chart of Patient enrollment. 196 PD patients were screened, and eventually 72 patients were enrolled. All of the enrolled patients received MRI and 67 of them finished cognitive tests

We screened 196 chronic peritoneal dialysis patients and enrolled 72 of them. All of them received brain magnetic resonance imaging screen and 67 of them completed the cognitive tests. One patient did not receive cognitive tests because of illiteracy, 1 patient died before the cognitive test because of heart failure, 1 patient was transferred to the other PD center, and 2 patients were admitted to the hospital because of peritonitis and hemorrhage of a polycystic kidney (Fig. 1). Questionnaires were also administered to gather the participant’s demographics and medical and dialysis histories.

### Demographic information and clinical features

Demographic information and clinical features are demonstrated in Table 1. In our PD patients, the average age was 56.2 yrs. nearly all of our patients had hypertension, but their blood pressures were controlled to an acceptable level. Additionally, 7.0% had a history of one episode of stroke based on patients’ self-reporting, and 80.0% of these strokes were ischemic strokes. Of note, 29.2% patients were receiving anti-platelet treatment.

**Table 1:** Demographic information and clinical features (*n* = 72) (mean ± sd)

Demographic information and clinical history	
Age (yr)	56.2 ± 16.0
Male (%)	37.5
Smoker (%)	16.7
Dialysis vintage (m)	41.2 ± 36.1
BMI (kg/m^2^)	22.9 ± 3.6
Average blood pressure (mmHg)	137.8/77.2 ± 21.6/13.6
Hypertension (%)	90.3
Hypertension duration (m, median (range))	95.8 (1.53–658.3);
Diabetes mellitus (%)	31.9
DM duration (m, median (range))	165.0 (48.3–462.5)
Atherosclerosis (%)	18.1
Atherosclerosis duration (m, median (range))	79.1 (4.2–164.2)
History of ischemia stroke (%)	5.6
History of hemorrhagic stroke (%)	1.4
Undergoing antiplatelet therapy^1^(%)	29.2
Patients on ACEI (%)	13.9
Patients on ARB (%)	36.1
Dialysis and laboratory evaluation	
Hemoglobin (g/L)	113.9 ± 13.7
Serum albumin (g/L)	35.4 ± 3.9
Serum creatinine (umol/L)	858.3 ± 271.1
Urea nitrogen (mmol/L)	18.0 ± 5.2
Serum uric acid (umol/L)	369.2 ± 60.0
Calcium and phosphamid product (mmol^2^/L^2^)	3.6 ± 1.0
Serum TCHO (mmol/L)	4.6 ± 1.0
Serum TG (mmol/L)	1.9 ± 1.2
hs CRP (mg/L)	6.4 ± 8.9
TSAT (%)	29.1 ± 12.0
iPTH (pg/ml)	359.7 ± 475.1
β2-MG (mg/L) (*n* = 66)	35.7 ± 14.1
Weekly total KT/Vurea^a^	2.2 ± 0.7
nPCR (g/kg/d)^b^	0.9 ± 0.25

^a^ total weekly Kt/Vurea(L/1.73 m^2^) = (Daily PD Kt/Vurea + (Daily RR Kt/Vurea) × 7 days. Daily PD Kt/Vurea = [dialysate urea concentration × 24-h peritoneal drain volume (L)/plasma urea concentrations]/ Vurea. Daily residual renal Kt Vurea = [Urine urea × 24-h urine volume (L)]/plasma urea concentrations]/ Vurea. Vurea: Adult male = 2.774–0.09516 x age (yr) + 0.1074 x Height (cm) + 0.3362 x weight (kg). Adult female = −0.2097 + 0.1069 x Height (cm) + 0.2466 x weight (kg)

^b^nPCR = 10.76[(Vd(L) xD_UN_(mmol/L) + Vu(L) xU_UN_(mmol/L))*14*2/1440(min) +1.46]/body weight. Vd, 24-h peritoneal drain volume; D_UN,_ the urea concentration in the pooled drained dialysate: Vu, 24-h urine volume; U_UN,_ the urea concentration in the urine.

^1^antiplatelet therapy including asprine and/or clopidogrel

18.1% patients reported themselves been diagnosed with atherosclerosis of carotid, coronary, or extremities arteries. Among these patients, 5 of them had carotid atherosclerosis. During a year after our study, 27 more patient received a carotid doppler and 22 of them were identified with carotid atherosclerosis, but only 2 of them were diagnosed with carotid stenosis.

Laboratory data and dialysis relative evaluations are also demonstrated in Table 1. The mean hemoglobin was 11.4 g/L and mean serum albumin level was 35.4 g/dL. The SCr was 857 umol/L. The average TCHO and TG were slightly elevated. The average hsCRP was 6.4 mg/L, nPCR was 0.9 g/kg/d and weekly Kt/V was 2.2.

### MRI results

**Fig. 2:**
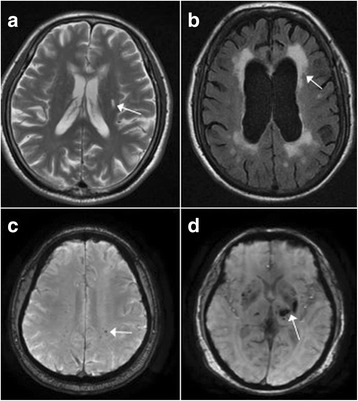
Typical images of different signs of CSVDs in PD patients. **a** Lacunar infarct, Insular subcortical, T2WI images; **b** Abnormal white matter hyperintensities, bilateral periventricular, T2-FLAIR images; **c** Microbleeds, bilateral parietal lobe white matter, SWI images; **d** Hematoma, bilateral basal ganglia and dorsal thalamus, SWI images

All participates received cerebral MR assessments. None had acute stroke lesions or hemorrhage. In MRI, 38.9% (28/72) of patients had lacunar infarcts (Fig. 2a), 48.6% (35/72) had abnormal WMHs (Fazekas scores > 2) (Fig. 2b),36.1% (26/72) of patients had microbleeds (Fig. 2c), and 4.2% (3/72) of patients had chronic intracerebral hemorrhage (Fig. 2d). Furthermore, 79.2% patients had periventricular WMHs (Fazekas scores > =1, only 9.7% P-WMH score > 2) and 81.9% patients had deep WMHs (Fazekas scores > =1, only 15.3% D-WMH score > 2).

### Comparison of characteristics across CSVD groups

In univariate models, we compared the demographic and clinical features between different signs of CSVD groups (Additional file 1: Table S1).

In our PD patients, patients with intracerebral hemorrhage had a lower nPCR level than patients without intracerebral hemorrhage (*p* = 0.014) and they had a higher percentage of patients with BMI greater than 25 kg/m^2^ (*p* = 0.0137).

Comparing patients with and without lacunar infarcts, patients with lacunar infarcts were older (*p* = 0.0001); their hsCRP levels were higher (*p* = 0.0057); and their nPCR (*p* = 0.0048), TSAT level (*p* = 0.0011), albumin (*p* = 0.0269), SCr (*p* = 0.0153) and urea nitrogen (*p* = 0.0146) were lower.

Compared with patients without abnormal WMHs, patients with abnormal WMHs were older (66.9 yr. vs 46.0 yr., *p* < 0.0001); their diastolic blood pressure was lower (*p* = 0.0336); hsCRP level was higher (*p* = 0.0149); albumin (*p* = 0.012) and SCr were lower (*p* = 0.055); and higher percentages of patients were receiving antiplatelet medicine (p = 0.004) and ARB (*p* = 0.0491).

Compared with patients without brain microbleeds, patients with microbleeds had a lower TSAT level (*p* = 0.008) and higher hsCRP level (*p* = 0.0649); also, there was a higher percentage of smokers (*p* = 0.0226) and more patients were receiving antiplatelet medicine (*p* = 0.0295).

In multivariable logistic regression analyses (Table 2), we focused on the effect of nPCR and hsCRP for different CSVD signs. In different models, we adjusted for the age, gender, smoking status, and other significant variables in univariate models. In all four regression models, nPCR was an independent risk factor for lacunar infarcts. There was a negative relative relationship between nPCR and lacunar infarcts, and there was a positive relative tendency between hsCRP and lacunar infarct. For abnormal WMHs, nPCR and hsCRP in univariate analyses had a similar influence on WMHs as for lacunar infarcts. However, after adjusting for age and gender, the influence reversed and lost statistical significance. For intracerebral hemorrhage and microbleeds, there was a similar tendency in the influence of nPCR and hsCRP on lacunar infarcts.

**Table 2:** Associations between nPCR/hsCRP and different kinds of CSVD^a^

Model	Intracerebral hemorrhage		lacunar infarcts		Abnormal WMH		Microbleeds	
	nPCR^b^	hsCRP^c^	nPCR	hsCRP	nPCR	hsCRP	nPCR	hsCRP
Model 1^d^	0.53 (0.29, 0.96)*	1.51 (0.62, 3.64)	0.76 (0.62, 0.93)*	1.65 (1.14, 2.4)*	0.93 (0.79, 1.11)	1.53 (1.08, 2.18)*	0.96 (0.81,1.15)	1.39 (0.98,1.99)
Model 2^e^	0.53 (0.29, 0.96)*	1.58 (0.58, 4.32)	0.75 (0.59, 0.95)*	1.29 (0.85, 1.97)	1.02 (0.79, 1.31)	0.91 (0.55, 1.52)	0.98 (0.82,1.18)	1.52 (1.00,2.33)*
Model 3^f^	0.51(0.28, 0.84)*	2.07 (0.68, 6.26)	0.67 (0.50, 0.89)*	1.23 (0.78, 1.92)	0.98 (0.76, 1.27)	0.92 (0.55, 1.55)	0.95 (0.78,1.15)	1.48 (0.95,2.30)
Model 4^g^	1.12 (0.45, 2.81)	*1.26 (0.22, 7.06)* ^*h*^	0.59 (0.39, 0.90)*	0.70 (0.35, 1.38)	1.07 (0.78, 1.47)	0.95 (0.52, 1.73)	0.95 (0.77,1.19)	1.29 (0.80,2.10)

^a^estimates were based on logistic regression; ^b^the unit of change is per log(nPCR)/10(g/kg/d); ^c^the unit of change is per log(hsCRP)(mg/L). ^d^only with nPCR/hsCRP; ^e^adjusted for age and gender; ^f^additionally adjusted diabetes and smoking status; ^g^additionally adjusted diabetes, smoking and significant variables in univariate analysis (for Intracerebral hemorrhage: BMI and nPCR; for lacunar infarcts; hsCRP, nPCR, TSAT, Scr, BUN and Alb; for abnormal WMH::hsCRP, Alb, diastolic blood pressure. ARB and antiplatelet medication for microbleeds: hsCRP, TSAT, antiplatelet medication). ^h^did not adjust for nPCR because of an abnormal coefficient. *p<0.05

In the subgroup analysis (Additional file 1: Table S3), for patients who were younger than 65 yrs. (*n* = 47), there was a similar influence of nPCR and hsCRP on four signs of CSVD in models 1, 2 and 3 in the whole cohort. However, limited by the sample size, the results did not reach statistical significance.

### Cognitive function evaluation

Sixty-seven of 72 participates participated in the cognitive function tests. The education level distribution was as follows: 9% (6/67) of participants received education less than 6 years, 25.4% (17/67) had education of 6–9 years, 25.4% (17/67) had education of 9–12 years, and 40.3% (27/67) had education > 12 years.

In the MMSE test, the average MMSE score was 27.6 ± 3.1. According to the MMSE score, 25% of patients could be diagnosed as abnormal. In the MoCA test, the average MoCA score was 21.7 ± 5.6. According to the MoCA score, 86.8% of patients can be diagnosed as abnormal. Possible factors that may have influence the MMSE/MoCA score were analyzed (Additional file 1: Table S2). The MMSE score was related to the age (*r* = −0.393, *P* = 0.0009), serum BUN (*r* = 0.262, *p* = 0.0317), TG level (*r* = −0.631, *p* = 0.0025), hsCRP (*r* = −0.345, *P* = 0.0042), nPCR (*r* = 0.319, *P* = 0.0083), and education level (*p* = 0.0006). The MoCA score was also related to the age (*r* = −0.534, *P* < 0.0001), SCr (*r* = 0.384, *p* = 0.0013), BUN(*r* = 0.280, *p* = 0.0219), TG level (*r* = −0.355, *p* = 0.0032), hsCRP (*r* = −0.315, *P* = 0.0095), nPCR (*r* = 0.342, *P* = 0.0047), and education level (*p* = 0.0022). Additionally, we found patients on ACEi had a higher MoCA score (*p* = 0.0286), but those on antiplatelet medicine had a lower MoCA score (*p* = 0.0591).

After adjusting for the age, gender, and smoking status as well as other significant variables in 4 different models, we found nPCR was an independent risk factor for decline of cognition, but hsCRP was not an independent risk factor (Table 3).

**Table 3:** Associations between nPCR/hsCRP and MMSE/MoCA^a^

	MMSE				MoCA			
	nPCR^b^		hsCRP^c^		nPCR		hsCRP	
Model	Coefficient (95%CI)	*p*	Coefficient (95%CI)	*p*	Coefficient (95%CI)	*p*	Coefficient (95%CI)	*p*
Model1^d^	0.15 (0.04, 0.25)	0.0083*	−0.03 (−0.05, 0.01)	0.0042*	0.40 (0.13, 0.68)	0.0047*	−0.07 (−0.12, −0.02)	0.0095*
Model2^e^	0.13 (0.03, 0.23)	0.0119*	−0.02 (−0.04, 0.01)	0.1509	0.36 (0.12, 0.59)	0.0033*	−0.02 (−0.07, 0.04)	0.5319
Model3^f^	0.14 (0.03, 0.24)	0.0107*	−0.02 (−0.04, 0.01)	0.1552	0.36 (0.12, 0.60)	0.0042*	−0.02 (−0.07, 0.04)	0.5765
Model4^g^	0.08 (−0.04, 0.18)	0.2109	0.01 (−0.02, 0.03)	0.5148	0.30 (0.03, 0.57)	0.0288*	0.05 (−0.01, 0.10)	0.1137

^a^estimates were based on general linear regression; ^b^the unit of change is per log(npcr) (g/kg/d); ^c^the unit of change is per log(hscrp)(mg/L). ^d^only with nPCR/hsCRP; ^e^additionally adjusted for age and gender;. ^f^additionally adjusted for diabetes and smoking status;. ^g^additionally adjusted significant variables in univariate analysis(Additional file 1: Table S2) (for MMSE, BUN, TG and hsCRP/nPCR; for MoCA, usage of ACEi, SCr, BUN, TG and hsCRP/nPCR) *p < 0.05

### Influence of different signs of CSVD in MRI to cognitive function

The influence of different signs of CSVD in MRI to cognitive function are shown in Table 4. By univariate analyses (Table 4), both the MMSE and MoCA scores were related to lacunar infarct lesions (*p* = 0.0007 and *p* < 0.0001, respectively) and abnormal WMHs (*P* = 0.0373 and *p* = 0.0104, respectively). After adjusting for different signs of CSVD (multiple- General linear model) (Table 4), MMSE and MoCA scores were only related to lacunar infarcts (*P* = 0.0132 and *p* = 0.00019, respectively).

**Table 4:** The influence of different CSVD signs on cognitive function

		MMSE(log)		MoCA(log)	
		Mean ± SD	*P*	Mean ± SD	*P*
Univariate analyses					
lacunar infarct	0 (*n* = 40)	3.35 ± 0.073	0.0007*	3.16 ± 0.20	<0.0001*
	1 (*n* = 27)	3.25 ± 0.16		2.86 ± 0.38	
Microbleed	0 (*n* = 43)	3.33 ± 0.13	0.1186	3.07 ± 0.32	0.1727
	1 (*n* = 24)	3.28 ± 0.12		2.96 ± 0.33	
chronic intracerebral hemorrhage	0 (*n* = 64)	3.31 ± 0.13	0.5324	3.04 ± 0.32	0.2300
	1 (*n* = 3)	3.27 ± 0.12		2.81 ± 0.43	
Abnormal WMH	0 (*n* = 35)	3.34 ± 0.09	0.0373*	3.13 ± 0.29	0.0104*
	1 (*n* = 32)	3.28 ± 0.15		2.93 ± 0.33	
Multivariate analyses					
lacunar infarct	0	3.36 ± 0.04	0.0132*	3.15 ± 0.10	0.0019*
	1	3.26 ± 0.04		2.84 ± 0.10	
Microbleed	0	3.31 ± 0.04	0.9904	2.97 ± 0.10	0.5463
	1	3.31 ± 0.04		3.02 ± 0.10	
chronic intracerebral hemorrhage	0	3.29 ± 0.02	0.7728	3.01 ± 0.10	0.5852
	1	3.32 ± 0.07		2.97 ± 0.09	
Abnormal WMH	0	3.32 ± 0.04	0.7297	2.97 ± 0.09	0.5443
	1	3.30 ± 0.04		3.02 ± 0.10	

*p < 0.05

## Discussion

The incidence of cerebrovascular accident in the dialysis population is 4–10 times higher than the general population [13] and non-dialysis CKD patients. The ten-year cerebrovascular cumulative incidence in dialysis patients can reach 20.9% [14]. Cerebrovascular disease is the third most common cause of death in dialysis patients [14–16].

CSVD including lacunar infarcts, WMHs, microbleeds and chronic Intracerebral hemorrhages. They are associated with cognitive function decline and could predict the prevalence of stroke. These lesions can only be detected by brain imaging because of the absence of clinical symptoms. In view of the serious cardio-cerebrovascular disease burden in ESRD patients, it is reasonable to deduce that there is a high risk of cerebral small vessel diseases in this population.

Most previous studies on cerebrovascular diseases in dialysis patients were based on “the clinically overt cerebrovascular accident”. We know little about the subclinical brain changes in dialysis patients. PD is not the mainstream dialysis modality in most of western countries. Because of those, there are very few data about the CSVD and its consequence in PD patients.

Our study, as we know, is the largest sample size study about the MRI evaluated CSVD and cognitive function in PD patients.

In this study, in PD patients, we found a high prevalence of CSVD, including lacunar infarct in 38.9%, cerebral microbleeds in 36.1%, abnormal WMHs in 48.6%, and intracerebral hemorrhage in 4.2%. As reported in the Northern Manhattan Study [2], the SBI prevalence was 17.7%, and 82.4% were less than 1 cm in size in the stoke free population; compared to these values, our PD cohort showed a much higher lacunar infarct prevalence. Compared to the 47% microbleeds prevalence in our HD cohort in a previous study [17], PD patients seems to have a lower risk for cerebral microbleeds than HD patients.

In our study, we also try to find a possible reason that may explain the high prevalence of CSVD in our PD cohort. In one-way analysis of variance, we found nPCR, which is used to assess dietary protein intake in dialysis patients and may reflect patient’s nutrition status, was lower in the lacunar infarct and intracerebral hemorrhage groups; hsCRP was higher in the lacunar infarct group (board lined), abnormal WMHs group and microbleed group (board lined). In addition to nPCR, other nutrition relative indexes, such as the serum albumin and SCr level, were lower in patients with lacunar infarcts or abnormal WMHs. These findings suggested that malnutrition and micro-inflammatory may be risk factors to or play an important role in the development of CSVD in PD patients.

In patients with end-stage renal disease (ESRD), chronic low grade inflammatory processes and malnutrition are common. Inflammation, protein-energy malnutrition and cardiovascular disease are interrelated and contributed to the high mortality in ESRD patients [18]. In peritoneal dialysis, some unique factors may also enhance chronic inflammation, such as episodes of peritonitis or peritoneal dialysis catheter-related infections and bioincompatible substances or endotoxins caused by conventional glucose-based dialysis fluids [19]. The term “malnutrition-inflammation-complex syndrome, MICS” indicated the possible close association between inflammation and malnutrition in dialysis patients [20]. Another term, “malnutrition-inflammation-atherosclerosis, MIA”, was used to emphasize the major consequence of MICS, atherosclerotic diseases. In uremia, malnutrition and inflammation may induce atherosclerosis through oxidative stress and endothelial dysfunction [21]. Based on these, it is reasonable to deduce that MICS in the ESRD population also played an important role in other vessel diseases, including the CSVD.

In our further multivariate logistic regression analyses, poor nPCR was the independent risk factor for lacunar infarcts in four different models, even after adjusted the hsCRP. However, in regression models, high hsCRP was not an independent risk factor for lacunar infarct. This means elevated hsCRP may be involved in the process from malnutrition to lacunar infarcts. For chronic Intracerebral hemorrhage and microbleeds, although the *p* value did not reach statistical significance in some models, it had a similar influence tendency of nPCR and hsCRP. For abnormal WMHs in logistic regression analyses, after adjusting for other factors in 4 models, the OR of nPCR was approximately 1, which means that the nutrition status may not be a major factor that induces abnormal WMHs in our PD patients. In model 1, the OR of hsCRP was 1.53, which also showed patients with elevated hsCRP had a higher risk of WMHs. In subgroup analysis, in patients younger than 65 yrs., we observed results in a similar direction as for the whole cohort; however, the sample size was limited. These results prompt further evaluation to investigate whether malnutrition is an initiating factor to the CSVD, and inflammation may be a downstream factor of the influence of malnutrition.

Cognitive impairment is an important CSVD sequence. It was considered that age, smoking, obesity, hypertension, diabetes mellitus and hypercholesterolemia may relate to cognitive impairment in the non-dialysis population. Stroke and silent brain infarction are also risk factors for subsequent cognitive decline [22].

There was a severe underestimation of our dialysis patients’ cognitive status by the clinicians In the DOPPS cohort (dialysis cohort), the recorded diagnosis of dementia was only 1–6% in different countries [23]. In PD patients, we know little about their cognitive status and the relationship between the CSVD and cognitive changes in this population.

In our PD cohort, with different cognitive functions tests, MMSE and MoCA, the prevalence of declined cognitive function was 25–86.8%. The average score of MMSE was 27.6 ± 3.1 and MoCA was 21.7 ± 5.6. Both were obviously lower than those of the similar age group (50–59 yr) of Beijing urban residents, whose average MMSE and MoCA scores were 29.79 ± 0.46 and 27.69 ± 1.57, respectively [12]. As the MoCA test is better at assessing the executive functions, complex visuospatial processing, and higher-level language abilities that can be mildly impaired in mild cognitive impairment [11], the MoCA test is more sensitive in evaluating PD patients’ cognition. Our patients’ MoCA test results indicated the characteristic of PD patients’ cognitive function decline is the non-amnestic component.

Similar to the general population, the age and education level are relative to the PD patients’ cognition. However, we also found that the hsCRP, nPCR, albumin (bordered), SCr, BUN, TG level and Angiotensin-Converting Enzyme Inhibitor (ACEi) medications were relative to PD patients’ cognition score. After adjusting for significant variables in univariate analyses, the nPCR was an independent risk factor for MMSE and MoCA. After adjusting for the nPCR, the hsCRP was not an independent risk factor for cognitive function. It indicated that malnutrition and micro-inflammation play important roles in cognitive function decline, and malnutrition may be an initiating factor that caused brain lesion and function changes in PD patients.

As for the relationship between cognition and CSVD, we found lacunar infarcts and WMHs are relative to our PD patients’ cognitive function, and these relationship were similar to those in the non-dialysis population [24, 25]. After multivariate analyses, only lacunar infarct was an independent risk factor. Other bleeding CSVD lesions, including microbleeds and chronic intracerebral hemorrhage, do not impact the cognitive status.

From the above relationship between nPCR and lacunar infarcts, nPCR and cognitive function, lacunar infarcts and cognitive, we could draw a conclusion that malnutrition might be the initial factor of the CSVD and the sequenced cognitive decline in PD patients.

This study had some limitations. Firstly, this was a cross-sectional study in which the causal relationship was not strong. Secondly, there were too many confounding factors to the MRI and cognitive changes, indicating the need for a much larger sample size. Thirdly, limited by our sample size, we chose MMSE/MoCA score which was a continuous variable instead of cognitive impairment which could be a dichotomous variable in our analysis, and we could not do a deeper analysis about the interaction between nPCR and hsCRP in CSVD and cognitive function.

## Conclusion

We demonstrated a high prevalence of CSVD and cognitive impairment in PD patients. Similar to non-dialysis population, in PD patients, among different signs of CSVD, lacunar infarct is most relevant to cognitive decline. Our novel observation revealed an association between malnutrition-inflammation and these cerebral outcomes, but these findings requires evaluation in a larger sample size population.

## Additional file

Additional file 1: Table S1. Differences in patients with and without different kinds of CSVD (*n* = 72)^a^. This table showed the comparison of the demographic and clinical features between subjects with or without of CSVD in univariate model. Four kinds of CSVD were compared separately. By these analyses, we got the primary idea about the possible factors that may be relative to CSVD and figured out the factors needed to be adjusted in the multivariate models. Table S2 ^a^ Factors that would influence cognitive functions. These were the analyses of the relationship between the demographic, clinical features and cognitive tests (the MMSE/MoCA test) scores in univariate model. By these analyses, we found out the factors which might influence the cognitive function and needed to be adjusted in the multivariate models. Table S3 Associations [OR(95%CIs)] between nPCR and hsCRP and different signs of CSVD in patients younger than 65 yrs. ^a^. These were sensitive analyses of the relationships between nPCR and hsCRP and different signs of CSVD in subjects who were younger than 65 yrs. . The results demonstrated similar tendency of influence of nPCR and hsCRP on four kinds of CSVD in model 1, 2 and 3 in the whole cohort. However, limited by the sample size, the results did not reach statistical significance. (DOCX 27 kb)

## Abbreviations

ACEi: Angiotensin-Converting Enzyme Inhibitor
BUN: Blood urea nitrogen
CI: Cognitive impairment
CI: Cognitive impairment
CKD: Chronic kidney disease
CSVD: Cerebral small vessel diseases
ESRD: End-stage renal disease
FLAIR: Fluid-attenuated inversion recovery
hsCRP: high sensitivity C reactive protein
iPTH: intact PTH
MIA: Malnutrition-inflammation-atherosclerosis
MICS: Malnutrition-inflammation-complex syndrome
MMSE: Mini Mental State Examination MoCA Montreal Cognitive Assessment
MR: Magnetic resonance
nPCR: Normalized protein catabolic rate
PD: Peritoneal dialysis
SBIs: Silent brain infarcts
SCr: Serum creatinine
SWI: Susceptibility weighted imaging
TCHO: Total cholesterol
TG: Triglyceride
TSAT: Transferrin saturation
WMHs: White matter magnetic resonance hyperintensities/white matter hyperintensities
