# Supplementary material for: Malnutrition-inflammation is a risk factor for cerebral small vessel diseases and cognitive decline in peritoneal dialysis patients: a cross-sectional observational study
Source: BMC Nephrol. 2017 Dec 20;18:366. doi: 10.1186/s12882-017-0777-1 (PMC5738894; doi:10.1186/s12882-017-0777-1)
Supplement: Additional file 1: Table S1. — Differences in patients with and without different kinds of CSVD (n = 72)a. This table showed the comparison of the demographic and clinical features between subjects with or without of CSVD in univariate model. Four kinds of CSVD were compared separately. By these analyses, we got the primary idea about the possible factors that may be relative to CSVD and figured out the factors needed to be adjusted in the multivariate models. Table S2 a Factors that would influence cognitive functions. These were the analyses of the relationship between the demographic, clinical features and cognitive tests (the MMSE/MoCA test) scores in univariate model. By these analyses, we found out the factors which might influence the cognitive function and needed to be adjusted in the multivariate models. Table S3 Associations [OR(95%CIs)] between nPCR and hsCRP and different signs of CSVD in patients younger than 65 yrs. a. These were sensitive analyses of the relationships between nPCR and hsCRP and different signs of CSVD in subjects who were younger than 65 yrs. . The results demonstrated similar tendency of influence of nPCR and hsCRP on four kinds of CSVD in model 1, 2 and 3 in the whole cohort. However, limited by the sample size, the results did not reach statistical significance. (DOCX 27 kb) [file 12882_2017_777_MOESM1_ESM.docx]

Additional files

| Additional file 1: Table S1 Differences in patients with and without different kinds of CSVD (n=72)^a^ | | | | | | | | | | | | |
| --- | --- | --- | --- | --- | --- | --- | --- | --- | --- | --- | --- | --- |
|  | Intracerebral hemorrhage | | | lacuna infracts | | | Abnormal WMH | | | microbleeds | | |
| Mean±SD | **no=69** | **yes=3** | **P** | **no=44** | **yes=28** | **P** | **no=37** | **yes=35** | **P** | **no=46** | **yes=26** | **P** |
| Age | 56.06±15.94 | 58.33±13.65 | 0.8088 | 50.70±15.22 | 64.71±12.68 | **0.0001*** | 46.00±13.58 | 66.89±9.68 | **<0.0001*** | 55.41±16.48 | 57.46±14.66 | 0.6002 |
| DBP | 77.03±13.11 | 81.33±27.21 | 0.5962 | 78.61±13.80 | 75.00±13.33 | 0.2763 | 80.51±14.53 | 73.71±11.85 | **0.0336*** | 76.47±13.90 | 78.53±13.33 | 0.5377 |
| TSAT(log) | 3.29＋0.44 | 3.06±0.55 | 0.3738 | 3.41±0.38 | 3.07±0.45 | **0.0011*** | 3.32±0.47 | 3.25±0.41 | 0.4973 | 3.38±0.41 | 3.10±0.44 | **0.0080*** |
| hsCRP(log) | 0.93±1.44 | 1.72±1.30 | 0.3573 | 0.60±1.39 | 1.54±1.33 | **0.0057*** | 0.57±1.38 | 1.39±1.39 | **0.0149*** | 0.73±1.58 | 1.38±1.01 | 0.0649 |
| nPCR(log) | -0.12±0.27 | -0.52±0.20 | **0.0140*** | -0.07±0.26 | -0.25±0.26 | **0.0048*** | -0.11±0.28 | -0.16±0.27 | 0.4288 | -0.13±0.29 | -0.16±0.26 | 0.6641 |
| ALB | 35.36±3.91 | 35.33±2.89 | **0.9780** | 36.16±3.30 | 34.11±4.38 | **0.0269*** | 36.46±3.69 | 34.20±3.74 | **0.0120*** | 35.76±4.01 | 34.65±3.54 | 0.2239 |
| **SCr** | 855.55±268.97 | 922.00±379.34 | 0.6808 | 919.50±252.04 | 762.18±276.54 | **0.0153*** | 917.81±283.88 | 795.43±245.50 | 0.0550 | 856.85±260.92 | 860.92±293.68 | 0.9517 |
| BUN | 18.11±5.25 | 14.69±4.14 | 0.2704 | 19.15±5.49 | 16.10±4.22 | **0.0146*** | 18.07±5.44 | 17.85±5.06 | 0.8593 | 18.27±5.58 | 17.41±4.58 | 0.951 |
| Bmi-group |  |  | **0.0137*** |  |  | 0.2798 |  |  | 0.1040 |  |  | 0.4104 |
| >25 | 15(21.74) | 3(100) |  | 9  (20.45) | 9  (32.14) |  | 6  (16.22) | 12  (34.29) |  | 10  (21.74) | 8  (30.77) |  |
| <25 | 54(78.26) | 0(0) |  | 35  (79.55) | 19  (67.86) |  | 31  (83.78) | 23  (65.71) |  | 36  (78.26) | 18  (69.23) |  |
| Smoking status |  |  | 1.00 |  |  | 0.1944 |  |  | 0.2145 |  |  | **0.0226*** |
| Non-smoker =0 | 57  (82.61) | 3  (100) |  | 39  (88.64) | 21  (75.00) |  | 33  (89.19) | 27  (77.14) |  | 42  (93.33) | 18  (69.23) |  |
| Smoker =1 | 12  (17.39) | 0(0) |  | 5  (11.36) | 7  (25.00) |  | 4  (10.81) | 8  (22.86) |  | 3  (6.67) | 8  (30.77) |  |
| Antiplatelet |  |  | 1.00 |  |  | 0.0622 |  |  | **0.0040*** |  |  | **0.0295*** |
| No =0 | 49  (71.01) | 2  (66.67) |  | 35  (79.55) | 16  (57.14) |  | 32  (86.49) | 19  (54.29) |  | 37  (80.43) | 14  (53.86) |  |
| Yes =1 | 20  (28.99) | 1  (33.33) |  | 9  (20.45) | 12  (42.86) |  | 5  (13.51) | 16  (45.71) |  | 9  (19.57) | 12  (46.15) |  |
| ARB |  |  | 1.00 |  |  | 1.000 |  |  | **0.0491*** |  |  | 0.4514 |
| no=0 | 44  (63.24) | 2  (66.67) |  | 28  (63.64) | 18  (62.07) |  | 28  (75.68) | 18  (51.43) |  | 31  (67.39) | 15  (57.69) |  |
| Yes=1 | 25  (36.76) | 1  (33.33) |  | 16  (36.36) | 11  (37.93) |  | 9  (24.32) | 17  (58.57) |  | 15  (32.61) | 11  (42.31) |  |
| *p<0.05,  ^a^ comparison of continuous variables between groups was performed with ANOVA, whereas the comparison of categorical variables was performed with the Chi-square test | | | | | | | | | | | | |

| Additional file 1: Table S2 ^a^ Factors that would influence cognitive functions | | | | | |
| --- | --- | --- | --- | --- | --- |
|  | MMSE(log) |  | | MoCA (log) |  |
| Demographic(n, mean+SD) |  | | p-value |  | p-value |
| **Gender** |  | 0.3867 | |  | 0.0982 |
| Male (n=25) | 3.33±0. 10 |  |  | 3.12±0.20 |  |
| Female (n=42) | 3.30±0.14 |  |  | 2.98±0.37 |  |
| **Education** |  | **0.0006*** | |  | **0.0022*** |
| <6 yr, low=1(n=6) | 3.19±0.11 |  |  | 2.62±0.37 |  |
| <12 yr, Middle=2(n=17) | 3.24±0.17 |  |  | 2.97±0.32 |  |
| 12~15 yr High=3(n=17) | 3.35±0.08 |  |  | 3.07±0.33 |  |
| >15 yr Undergraduate=4(n=27) | 3.36±0.07 |  |  | 3.14±0.24 |  |
| **Smoking status** |  | 0.6170 | |  | 0.3953 |
| Smoker =1(n=12) | 3.31±0.13 |  |  | 3.10±0.26 |  |
| Non-smoker =0(55) | 3.33±0.11 |  |  | 3.02±0.34 |  |
| **Diabetes** |  | 0.4704 | |  | 0.1710 |
| Yes=1(n=21) | 3.30±0.17 |  |  | 2.95±0.44 |  |
| No=0(n=46) | 3.32±0.10 |  |  | 3.07±0.25 |  |
|  | | | | | |
| **Clinical evaluation** | **(correlation, r)** | | |  |  |
| Age | -0.39318 | **0.0009*** | | -0.53403 | **<0.0001*** |
| **SBP** | 0.04961 | 0.6902 | | 0.03154 | 0.8000 |
| **DBP** | 0.17508 | 0.1565 | | 0.20298 | 0.0969 |
| Alb | 0.15985 | 0.1963 | | 0.2237 | 0.0688# |
| SCr | 0.17678 | 0.1524 | | 0.38453 | **0.0013*** |
| BUN | 0.26279 | **0.0317*** | | 0.27960 | **0.0219*** |
| UA | 0.01079 | 0.9309 | | 0.09648 | 0.4374 |
| β2-MG | -0.13858 | 0.2868 | | 0.06351 | 0.6268 |
| CHO | -0.06091 | 0.6244 | | -0.00110 | 0.9296 |
| HDLC | 0.16552 | 0.1807 | | 0.19528 | 0.1133 |
| LDLC | 0.16434 | 0.839 | | 0.15462 | 0.2116 |
| TG(log) | -0.6306 | **0.0025*** | | -0.35523 | **0.0032*** |
| **Hgb** | 0.04062 | 0.7442 | | -0.00264 | 0. 9831 |
| TSAT(log) | 0.20228 | 0.1007 | | 0.22103 | 0.0723# |
| Weekly Kt/V(log) | 0.00110 | 0.9930 | | -0.04157 | 0.7384 |
| **hsCRP(log)** | -0.34514 | **0.0042*** | | -0.31475 | **0.0095*** |
| **nPCR(log)** | 0.31976 | **0.0083*** | | 0.34157 | **0.0047*** |
|  |  |  | |  |  |
| **Medication (n, mean±SD)** |  |  | |  |  |
| Antiplatelet |  | 0.1038 | |  | 0.0591# |
| No =0(n=48) | 3.33±0.10 |  |  | 3.08±0.28 |  |
| Yes =1(n=19) | 3.27±0.17 |  |  | 2.91±0.39 |  |
| antihp_acei（ACEI） |  | 0.0811 | |  | **0.0286*** |
| No =0(n=59) | 3.30±0.13 |  |  | 3.00±0.34 |  |
| Yes =1(n=9) | 3.38±0.03 |  |  | 3.25±0.09 |  |
| antihp_arb (ARB) |  | 0.9947 | |  | 0.5843 |
| no=0(n=42) | 3.31±0.11 |  |  | 3.01±0.33 |  |
| Yes=1(n=25) | 3.31±0.16 |  |  | 3.06±0.32 |  |
| ^a^ comparison of continuous variables between groups was performed with ANOVA, whereas the comparison of categorical variables was performed with the Chi-square test | | | | | |

| Additional file 1: Table S3 Associations [OR(95%CIs)] between nPCR and hsCRP and different signs of CSVD in patients younger than 65 yrs ^a^ | | | | | | | | | | | | | | | | |
| --- | --- | --- | --- | --- | --- | --- | --- | --- | --- | --- | --- | --- | --- | --- | --- | --- |
| Model | Intracerebral hemorrhage | | | | Lacuna infarcts | | | | Abnormal WMH | | | | Microbleeds | | | |
|  | nPCR^b^ | P | hsCRP^c^ | P | nPCR | P | hsCRP | P | nPCR | P | hsCRP | P | nPCR | P | hsCRP | P |
| **Model 1^d^** | 0.534(0.277,1.028) | 0.061# | 1.898(0.605,5.957) | 0.272 | 0.838(0.639,1.099) | 0.201 | 1.043(0.627,1.736) | 0.872 | 1.050(0.821, 1.342) | 0.697 | 1.041(0.642,1.686) | 0.871 | 0.893(0.706,1.129) | 0.343 | 1.302 (0.823,2.060) | 0.260 |
| **Model 2^e^** | 0.528(0.272,1.026) | 0.060# | 2.114(0.577,7.748) | 0.259 | 0.818(0.613,1.091) | 0.171 | 1.043 (0.626,1.738) | 0.871 | 1.071(0.819,1.403) | 0.615 | 1.044 (0.643,1.695) | 0.863 | 0.929 (0.721,1.196) | 0.567 | 1.337 (0.835,2.142) | 0.226 |
| **Model 3^f^** | 0.572(0.302,1.083) | 0.086# | 2.196(0.595,8.106) | 0.337 | 0.765(0.555,1.054) | 0.102# | 1.068 (0.622,1.835) | 0.810 | 1.085 (0.822,1.432) | 0.567 | 1.063 (0.650,1.736) | 0.808 | 0.869 (0.660,1.143) | 0.315 | 1.348 (0.828,2.193) | 0.229 |
| ^a^ estimates were based on logistic regression; ^b^ the unit of change is per log(nPCR)/10(g/kg/d); ^c^ the unit of change is per log(hsCRP)(mg/L)  ^d^ only with nPCR/hsCRP; ^e^ adjusted for age and gender; ^f^ additionally adjusted smoking status | | | | | | | | | | | | | | | | |
